# Supplementary material for: Multi-Omics Analysis Reveals Synergistic Enhancement of Nitrogen Assimilation Efficiency via Coordinated Regulation of Nitrogen and Carbon Metabolism by Co-Application of Brassinolide and Pyraclostrobin in Arabidopsis thaliana
Source: Int J Mol Sci. 2023 Nov 17;24(22):16435. doi: 10.3390/ijms242216435 (PMC10671621; doi:10.3390/ijms242216435)
Supplement: Supplementary file 1 [file ijms-24-16435-s001.zip › ijms-2711671-SI.pdf]

Supporting information for：

Multi-omics analysis reveals synergistic enhancement of N assimilation efficiency via coordinated regulation of nitrogen and carbon metabolism by co-application of brassinolide and pyraclostrobin in *Arabidopsis thaliana*

Ya-Qi An[1]*, De-Jun Ma[1], and Zhen Xi[1,2,3]*

1 State Key Laboratory of Elemento-Organic Chemistry, and Department of Chemical Biology, National Pesticide Engineering Research Center, College of Chemistry, Nankai University, Tianjin 300071, P. R. China.

2 Frontiers Science Center for New Organic Matter, Nankai University, Tianjin 300071, P. R. China.

3 Collaborative Innovation Center of Chemical Science and Engineering, Tianjin 300071, P. R. China.

*corresponding author: E-mail: 1120180343@mail.nankai.edu.cn (Ya-Qi An), zhenxi@nankai.edu.cn (Zhen Xi), Tel: +86 022-23504782. Fax: +86 022-23504782.

Contents:

Table S1. The prime list for qRT-PCR. 2

Figure S1. The dry weight of leaves per plant. 3

Figure S2. qRT-PCR confirmation of RNA-seq data 3

Figure S3: The number and family of the transcription factors differentially regulated by the Pyr + BL rather than by BL or Pyr. 4

Figure S4: The identification of differentially accumulated amino acids and their derivatives based on metabolomic analysis. 5

Table S4: The analysis of the KEGG pathway enriched by DAMs versus the untreated group based on metabolomic analysis. 6

Figure S5. Heatmap showing the correlation between DEGs and DAMs among the 4 groups 8

Figure S6. The shared KEGG pathways enriched both by DEGs and DAMs among the 4 groups 9

Figure S7. Heatmap showing the correlation between the genes and metabolites that are specifically differentially regulated by the BL+Pyr treatment rather than by the BL or Pyr alone treatment. 10

Table S1. The prime list for qRT-PCR.

gene	primer sequence
ACTIN2-qRT-F	TGTGCCAATCTACGAGGGTTT
ACTIN2-qRT-R	TTTCCCGCTCTGCTGTTGT
NIA1-qRT-F	AACGCAGGTACAGATTGC
NIA1-qRT-R	GGAAGAGTCGTAGCCAGT
NIA2-qRT-F	CACGTCCCTAAAGCCCAA
NIA2-qRT-R	CCATGTTCTGTTCCTTACGG
NIR1-qRT-F	AGCGATTCCTCTTGATGC
NIR1-qRT-R	GTTCGTCGATAAGCCACA
NPF6.3-qRT-F	GAAGGCTAGTGTCTCGGGTT
NPF6.3-qRT-R	ACAAGGACCGTCACAGCTAA
GLU1-qRT-F	CTTCTGCATGGGCGACGATA
GLU1-qRT-R	CCTAAGGGGGTCAATGGCAG
LBD 37-qRT-F	TGCTTTGTTTCAGTCGTGCT
LBD 37-qRT-R	TGCTCCGTTAACTGGATTGACA
NLP7-qRT-F	GAGTTTGCCCGACGACAATGAAG
NLP7-qRT-R	GGCCTCCATCAGTACCTTGAACAG
GLN1-1-qRT-F	GTCATGTGCGATGCGTACAC
GLN1-1-qRT-R	TGATCCCAGCGTATAAGCAGG
GDH2-qRT-F	GGATTCATGTGGGAAGAGGA
GDH2-qRT-R	GCGACTCGGTTAACTCCAAG
RAV1-qRT-F	TTCGAAATCTGAGATCGTTG
RAV1-qRT-R	TACTCAACCCCGACGTTAACAA

Figure S1. The dry weight of leaves per plant.

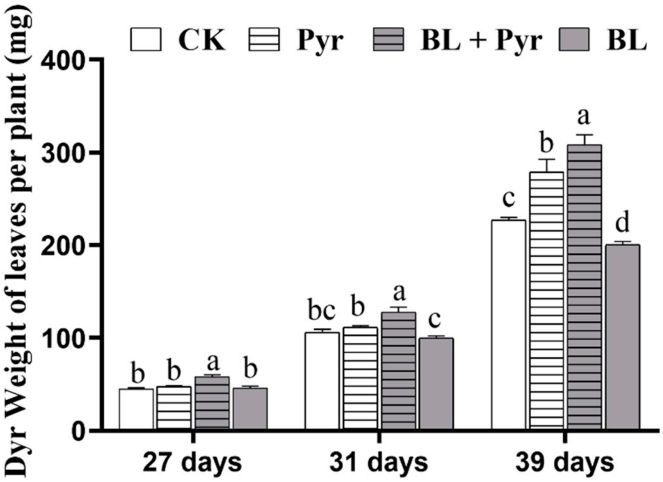

Note: Leaves were sampled at 27-, 31- and 39-day-old seedings in the untreated group (CK), Pyr group (treated with 3 μM pyraclostrobin), BL+Pyr group (treated with 1 μM BL and 3 μM pyraclostrobin), and BL group (treated with 1 μM BL), respectively. Data was presented as the mean ± SD of three separate replicate experiments. Different letters indicate significant differences between the 4 treatments based on t-test comparisons at $p<0.05$.

Figure S2. qRT-PCR confirmation of RNA-seq data.

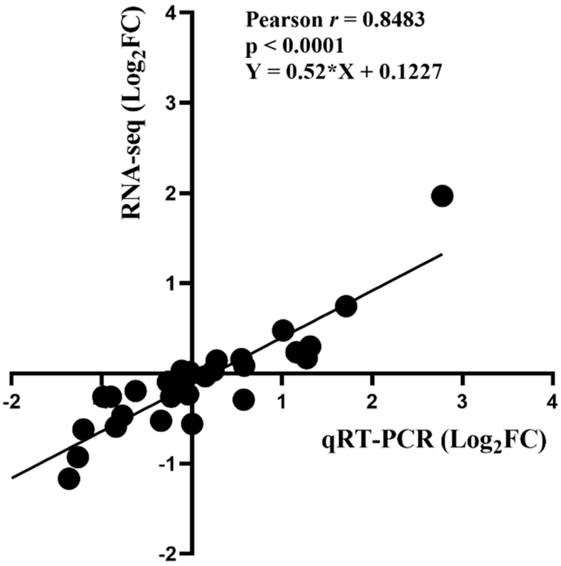

Note: The expression levels of ten nitrogen metabolism-related DEGs (LBD37, NLP7, RAV1, NPF6.3, GDH2, GLN1-1, GLU1, NIR1, NIA2, and NIA1) were analyzed by qRT-PCR. Correlation analysis between Log_2(fold change) data from RNA-seq (y-axis) and qRT-PCR (x-axis) of the BL+Pyr, BL, and Pyr groups versus the untreated group for these ten genes was carried out. Each data point was the mean of three biological replicates.

Figure S3: The number and family of the transcription factors differentially regulated by the Pyr + BL rather than by BL or Pyr.

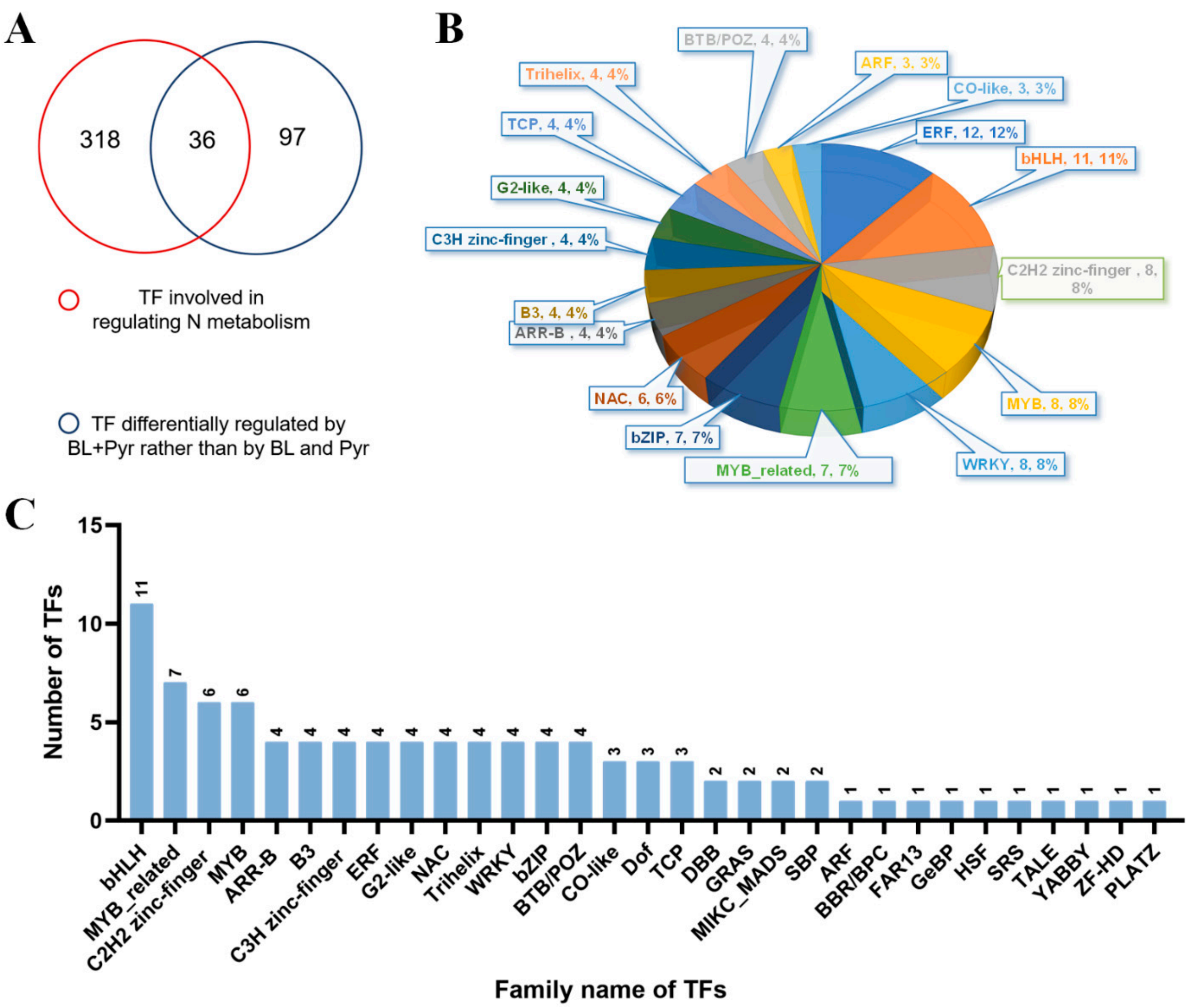

Notes: A: Venn diagrams showing the overlap and non-overlap between BL+Pyr uniquely differentially regulated transcription factors in this study and nitrogen metabolism-related transcription factors in previous literature; C: Pie chart showing the number and family of those 36 overlapping transcription factors between BL+Pyr uniquely differentially regulated transcription factors in this study and nitrogen metabolism-related transcription factors in previous literature; C: The number and family of those 97 non-overlapping transcription factors between BL+Pyr uniquely differentially regulated transcription factors in this study and nitrogen metabolism-related transcription factors in previous literature.

Figure S4: The identification of differentially accumulated amino acids and their derivatives based on metabolomic analysis.

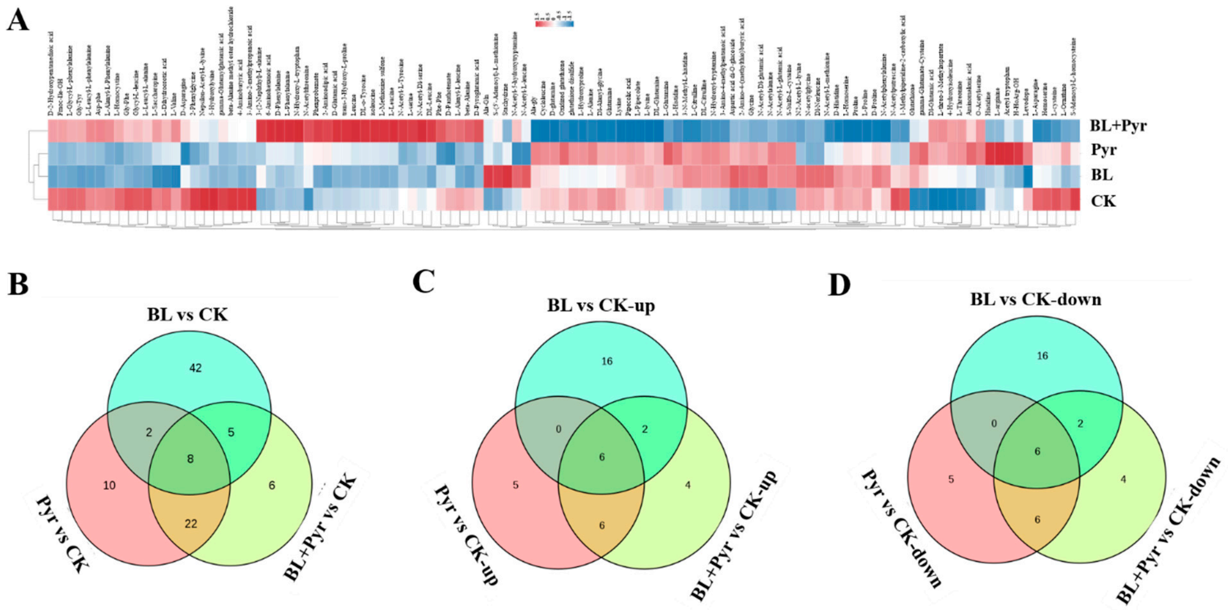

Notes: A: Hierarchical cluster analysis according to the accumulation of amino acids and their derivatives in the leaves; B-D: Venn diagrams showing the overlapping and non-overlapping of differentially accumulated amino acids and their derivatives between the untreated group and the 3 groups (BL+Pyr, BL, and Pyr). Leaves were collected at 31-day-old seedings in the untreated group (CK), Pyr group (treated with 3 μM pyraclostrobin), BL+Pyr group (treated with 1 μM BL and 3 μM pyraclostrobin), and BL group (treated with 1 μM BL), respectively.

Table S4: KEGG pathway enrichment analysis for DAMs of the 3 treatments.

KEGG	Pyr vs CK	Pyr+BL vs CK	BL vs CK
Photosynthesis		✓	
Inositol phosphate metabolism		✓	
Vitamin B6 metabolism		✓	
Monoterpenoid biosynthesis		✓	
Lysine biosynthesis	✓		
Pyruvate metabolism	✓		
Flavonoid biosynthesis	✓		
2-Oxocarboxylic acid metabolism	✓		
Fatty acid degradation	✓		
Pantothenate and CoA biosynthesis	✓		
Betalain biosynthesis	✓		
Taurine and hypotaurine metabolism	✓		
Arginine and proline metabolism			✓
Phenylalanine, tyrosine and tryptophan biosynthesis			✓
Selenocompound metabolism			✓
Plant hormone signal transduction			✓
Aminoacyl-tRNA biosynthesis			✓
Tropane, piperidine and pyridine alkaloid biosynthesis			✓
Glucosinolate biosynthesis			✓
Glutathione metabolism			✓
Phenylpropanoid biosynthesis			✓
Caffeine metabolism			✓
Sulfur metabolism			✓
Sulfur relay system			✓
Pentose phosphate pathway	✓	✓	✓
Carbon metabolism	✓	✓	✓
Porphyrin and chlorophyll metabolism	✓	✓	✓
ABC transporters	✓	✓	✓
Sphingolipid metabolism	✓	✓	✓
Biosynthesis of amino acids	✓	✓	✓
Isoquinoline alkaloid biosynthesis	✓	✓	✓
Histidine metabolism	✓	✓	✓
Anthocyanin biosynthesis	✓	✓	✓
Linoleic acid metabolism	✓	✓	✓
Phosphonate and phosphinate metabolism	✓	✓	✓
Tyrosine metabolism		✓	✓
Monobactam biosynthesis		✓	✓
Carbon fixation in photosynthetic organisms		✓	✓
Biosynthesis of unsaturated fatty acids		✓	✓
alpha-Linolenic acid metabolism		✓	✓

Glycine, serine and threonine metabolism		✓	✓
Nicotinate and nicotinamide metabolism		✓	✓
Riboflavin metabolism	✓	✓	
Starch and sucrose metabolism	✓	✓	
Citrate cycle (TCA cycle)	✓	✓	
C5-Branched dibasic acid metabolism	✓	✓	
Alanine, aspartate and glutamate metabolism	✓	✓	
Glyoxylate and dicarboxylate metabolism	✓	✓	
Arginine biosynthesis	✓	✓	
Purine metabolism	✓	✓	
Oxidative phosphorylation	✓	✓	
Thiamine metabolism	✓	✓	
Butanoate metabolism	✓	✓	
Ascorbate and aldarate metabolism	✓	✓	
Nitrogen metabolism	✓	✓	
Pentose and glucuronate interconversions	✓	✓	
Pyrimidine metabolism	✓	✓	
Galactose metabolism	✓	✓	
Valine, leucine and isoleucine biosynthesis	✓		✓
Cyanoamino acid metabolism	✓		✓
beta-Alanine metabolism	✓		✓
Valine, leucine and isoleucine degradation	✓		✓
Cysteine and methionine metabolism	✓		✓
Flavone and flavonol biosynthesis	✓		✓

Notes: The "✓" represented that the DAMs annotated to this pathway were identified in this group.

Leaves were collected at 31-day-old seedings in the untreated group (CK), Pyr group (treated with 3 μM pyraclostrobin), BL+Pyr group (treated with 1 μM BL and 3 μM pyraclostrobin), and BL group (treated with 1 μM BL), respectively.

Figure S5. Heatmap showing the correlation between DEGs and DAMs among the 4 groups

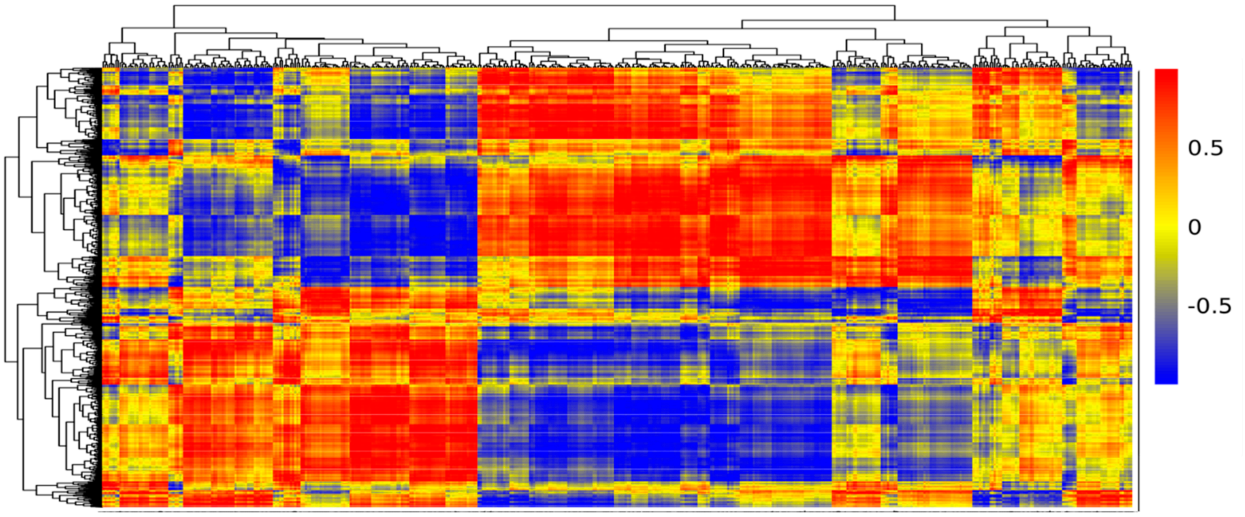

Notes: Each cell in the heatmap represents the correlation coefficient between a specific DEG and DAM. The color intensity of each cell reflects the strength of the correlation, with darker colors indicating stronger correlations. Red color means positive correlation and blue color means negative correlation. The 4 groups included the untreated group (CK), Pyr group (treated with 3 μM pyraclostrobin), BL+Pyr group (treated with 1 μM BL and 3 μM pyraclostrobin), and BL group (treated with 1 μM BL), respectively.

Figure S6. The co-enrichment analysis conducted on DEGs and DAMs among the 4 groups within KEGG pathways.

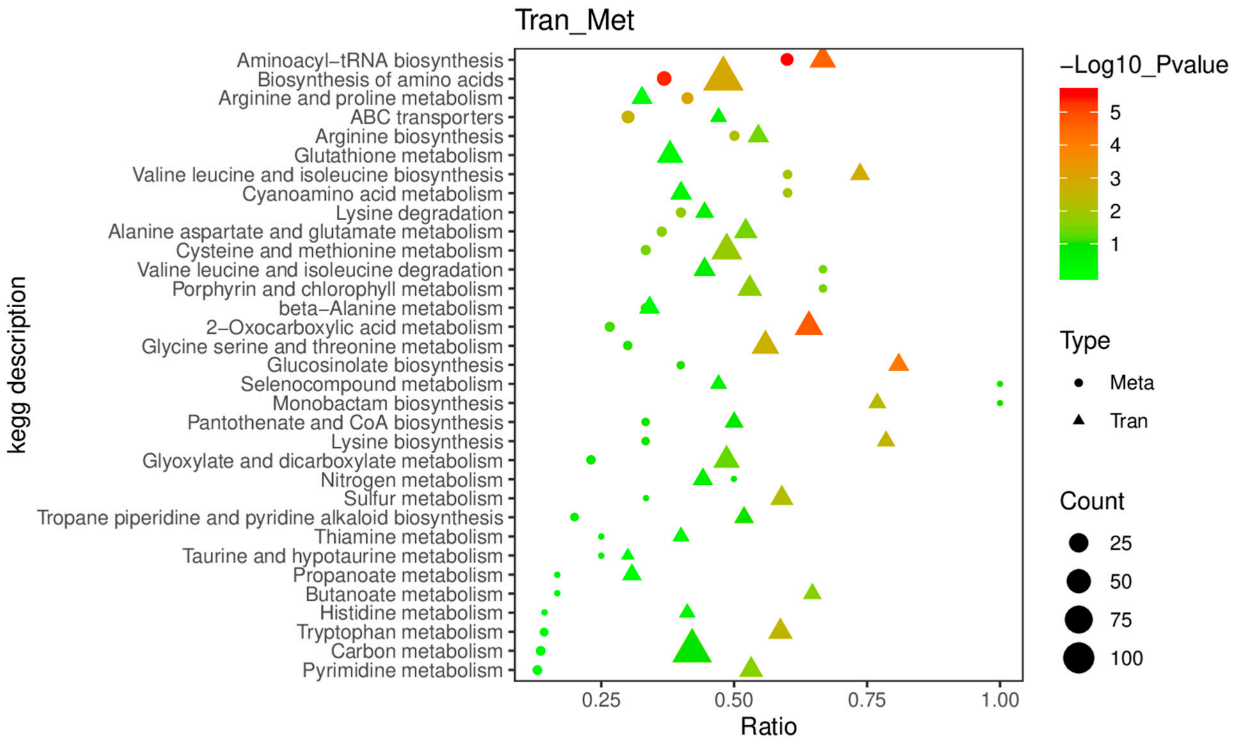

Notes: The enriched KEGG pathways are plotted along the X-axis, while the Y-axis signifies the enrichment factor. Triangles represent DEGs, circles represent DAMs. The bubble size correlates with the count of DAMs or DEGs, and the bubble color indicates the -log10 (P-value), providing a measure of statistical significance. The 4 groups included the untreated group (CK), Pyr group (treated with 3 μM pyraclostrobin), BL+Pyr group (treated with 1 μM BL and 3 μM pyraclostrobin), and BL group (treated with 1 μM BL), respectively.

Figure S7. Heatmap showing the correlation between the genes and metabolites that are specifically differentially regulated by the BL+Pyr treatment rather than by the BL or Pyr alone treatment.

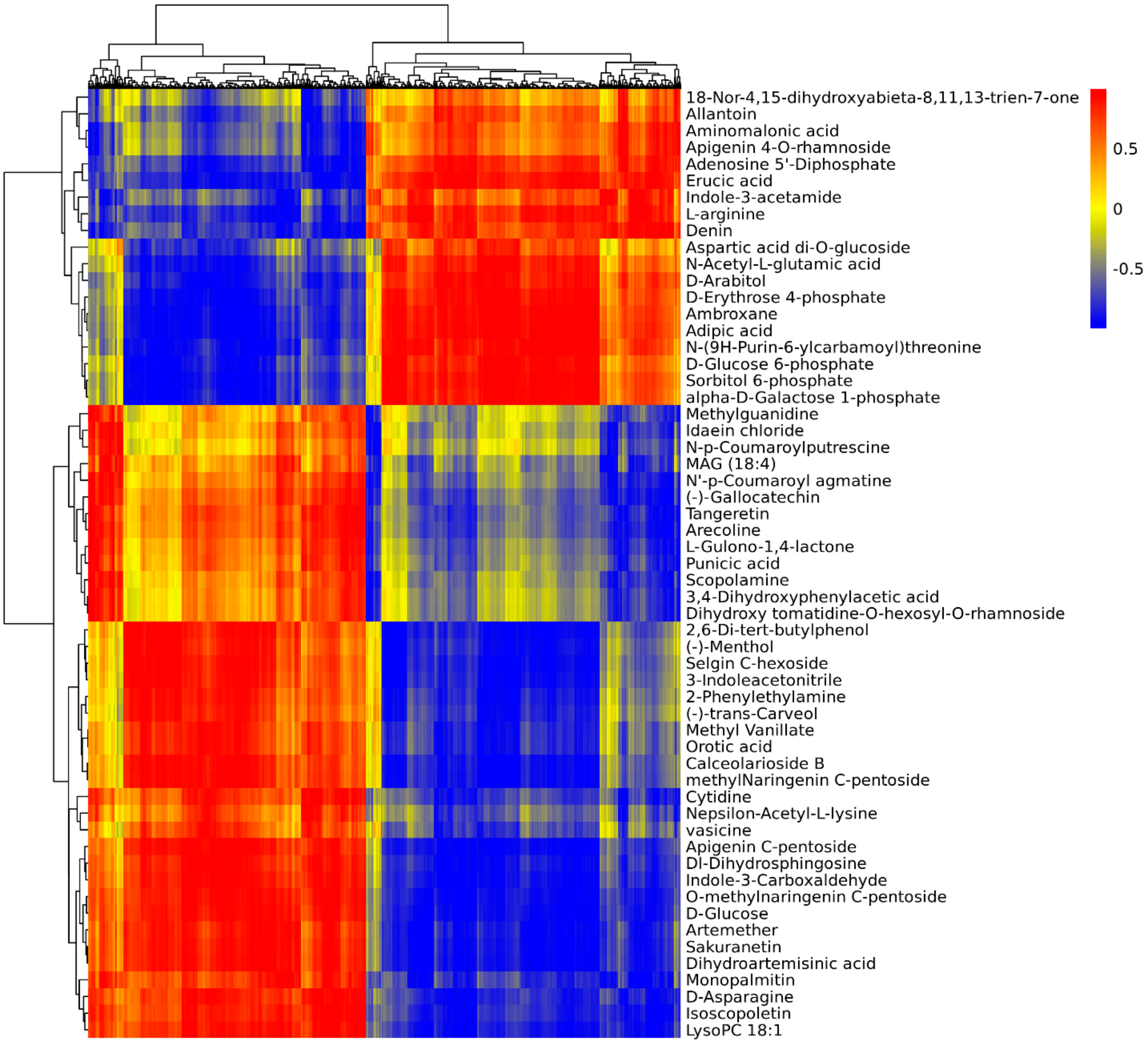

Notes: Each cell in the heatmap represents the correlation coefficient between a specific DEG and DAM. The color intensity of each cell reflects the strength of the correlation, with darker colors indicating stronger correlations. Red color means positive correlation and blue color means negative correlation.

The following tables are provided in a separate supplementary document (Supplyment Table 2, 3, 5, 6, 7.xlsx)due to space constraints.

Table S2: The list of significantly enriched GO terms of DEGs induced by BL+Pyr rather than by BL or Pyr;

Table S3: The list of significantly enriched KEGG pathways of DEGs induced by BL+Pyr rather than by BL or Pyr;

Table S5: Pearson correlation analysis between DEGs and DAMs among the 4 groups;

Table S6: Pearson correlation analysis between DEGs and DAMs unique to BL+Pyr;

Table S7: The list of BL+Pyr-specific DEGs and DAMs that share the KEGG pathway.
